# Androgen Receptor Activation in Glioblastoma Can Be Achieved by Ligand-Independent Signaling through EGFR—A Potential Therapeutic Target

**DOI:** 10.3390/ijms222010954

**Published:** 2021-10-11

**Authors:** Nomi Zalcman, Mijal Gutreiman, Tal Shahar, Michael Weller, Iris Lavon

**Affiliations:** 1 Molecular Neuro-Oncology Laboratory, Leslie and Michael Gaffin Center for Neuro-Oncology, Agnes Ginges Center for Human Neurogenetics, Neurology Department, Hadassah Hebrew University Medical Center, P.O. Box 12000, Jerusalem 91120, Israel; nomi.zlcman@mail.huji.ac.il (N.Z.); tmprober@gmail.com (M.G.); 2 The Laboratory for Molecular Neuro-Oncology, Department of Neurosurgery, Shaare Zedek-Hebrew University Medical Center, P.O. Box 3235, Jerusalem 9103102, Israel; tals@szmc.org.il; 3 Laboratory for Molecular Neuro-Oncology, Department of Neurology, University Hospital, University of Zurich, CH-8091 Zurich, Switzerland; michael.weller@usz.ch

**Keywords:** glioblastoma, androgen receptor (AR), epidermal growth factor receptor (EGFR), protein kinase B (AKT), AR antagonist

## Abstract

Androgen receptor (AR) is a ligand-mediated transcription factor that belongs to the superfamily of steroid receptors. AR is overexpressed in most glioblastomas and is a potential therapeutic target. In prostate and breast cancers, AR activation can be achieved also by a ligand-independent signaling through receptor tyrosine kinases such as epidermal growth factor receptor (EGFR). Considering its major role in glioblastoma, we explored whether EGFR is involved in AR signaling in this tumor. Analysis of mRNA expression in 28 glioblastoma samples with quantitative real-time reverse-transcription polymerase chain reaction revealed a positive and significant correlation between AR and EGFR mRNA expression levels (R = 0.47, *p* = 0.0092), which was validated by The Cancer Genome Atlas dataset (*n* = 671) analysis (R = 0.3, *p* = 0.00006). Using Western blotting and immunofluorescence staining, we showed that the transduced overexpression of EGFR or its variant EGFRvIII in the U87MG cells induced AR protein overexpression and nuclear translocation and Protein kinase B (AKT) S473 and AR S210/213 phosphorylation. The EGFR kinase inhibitor afatinib and the AKT inhibitor MK2206 reduced AR nuclear translocation. Afatinib diminished AKT phosphorylation at 30 min and 6 h in the EGFR- and EGFRvIII-overexpressing cells, respectively, and decreased AR phosphorylation in EGFR-overexpressing cells at 4 h. Afatinib or MK2206 combination therapy with the AR antagonist enzalutamide in the EGFR and EGFRvIII-overexpressing cells had synergistic efficacy. Our findings suggest that EGFR signaling is involved in AR activation in glioblastoma and buttresses the concept of combining an EGFR signaling inhibitor with AR antagonists as a potential glioblastoma treatment.

## 1. Introduction

Glioblastoma is the most common and most aggressive primary brain tumor, with a very poor prognosis. One of the most common genetic aberrations associated with glioblastoma is epidermal growth factor receptor (EGFR) amplification [1]. EGFR is overexpressed in approximately 60% of tumors. Half of the EGFR-amplified glioblastomas carry a tumor-specific deletion variant (EGFRvIII), which is characterized by the in-frame deletion of exons 2–7, resulting in constitutive EGFR activation [2]. EGFR belongs to the four-member of Human Epidermal Growth Factor Receptor (HER); receptor tyrosine kinase (RTK) family: EGFR (HER1), HER2, HER3, and HER4. EGFR signaling results in downstream signaling pathway activation, e.g., the phosphoinositide-3 kinase (PI3K)–AKT–mammalian target of rapamycin (mTOR) pathway. In glioblastoma, this pathway downstream of EGFR is often hyperactivated not only by overexpression or activating *EGFR* mutations but also by Phosphatase and tensin homolog (*PTEN*) deletion or downregulation, which antagonizes PI3K activity [3]. Drugs targeting EGFR, e.g., afatinib, which irreversibly inhibits HER2, HER4, and EGFR kinases [4]; Erbitux, a chimeric (mouse/human) anti-EGFR monoclonal antibody (mAb) [5]; or erlotinib that binds reversibly to the receptor’s ATP binding site, have activity in different tumor types. However, glioblastoma clinical studies have not shown any benefit of EGFR kinase inhibitors or mAbs, typically in patient populations with glioblastoma unselected for molecular marker profiles [6,7].

Typically, the androgen receptor (AR) functions as a steroid hormone-activated transcription factor. Upon binding androgens such as testosterone or dihydrotestosterone (DHT), AR is released from its chaperone and translocated to the nucleus, where it binds to the androgen response element in the promoter and stimulates the transcription of androgen-responsive genes [8].

AR plays an essential role in prostate cancer [9,10,11] and modulates the expression of cell cycle-, survival-, and growth-regulating proteins [12].

AR is amplified at DNA and RNA levels; 56% of glioblastomas express AR protein [13]. We recently showed that AR protein expression could be detected in glial tumors in real time by positron emission tomography/computed tomography scanning 16β-18F-fluoro-5α-dihydrotestosterone [14]. Pharmacological inhibition of AR and *AR* genetic silencing in glioblastoma cell lines induced cell death, while AR antagonists significantly decreased human glioblastomas implanted subcutaneously [13].

Ligand-independent AR phosphorylation and the subsequent activation, seen primarily in prostate cancers and breast and other cancer types, can be achieved through several RTKs, including EGFR family members [15,16,17,18]. These AR-independent pathways can promote cancer cell survival and growth and appear to be a significant androgen-independent driver of AR-regulated gene expression [19,20,21].

Considering the low androgen levels in the adult brain [22] compared to prostate cancer, we speculated that in contrast to prostate cancer, where androgens are the main drivers of cell proliferation [8], AR-mediated glioblastoma cell proliferation involves androgen-independent AR signaling.

Based on this and the significant role of EGFR in glioblastoma, we explored whether AR activation in glioblastoma is achieved through EGFR signaling.

## 2. Results

### 2.1. AR Expression in Glioblastoma Samples Correlates Significantly with EGFR Expression Levels

**Figure 1:**
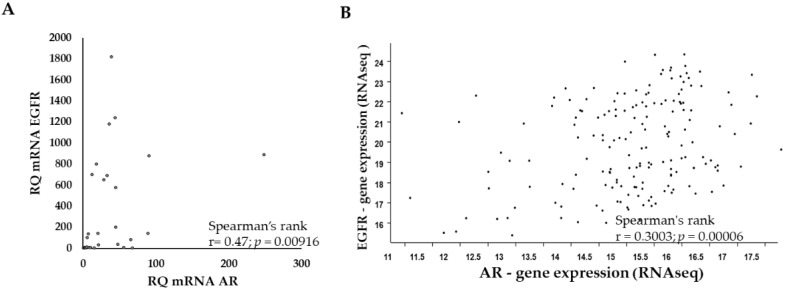
AR and EGFR expression levels are highly correlated in glioblastoma. (**A**) Spearman correlation analysis of qPCR results of AR versus EGFR mRNA expression following normalization to HPRT in 28 glioblastoma tumor samples. *Y*-axis, relative quantification (RQ) of EGFR; *X*-axis, the RQ of AR. (**B**) Spearman correlation analysis of AR and EGFR RNA sequencing (RNA-seq) results from glioblastoma samples (*n* = 671) of the National Cancer Institute Genomic Data Commons TCGA following normalization by log2 [fragment per kilobase per million reads map (fpkm-uq + 1)]. Each dot on the graph represents one tumor sample.

Considering the conflicting results on the correlation between EGFR and AR expression in various cancer types, we explored their expression by reverse transcription quantitative polymerase chain reaction (RT-qPCR) in glioblastoma samples from 28 patients. The AR and EGFR expression levels in the samples correlated positively (R = 0.47; *p* = 0.00916) (Figure 1A). The EGFR and AR expression data obtained by RNA sequencing (RNA-seq) of The Cancer Genome Atlas (TCGA) dataset (*n* = 671) (Figure 1B) yielded similar results (R = 0.3; *p* = 0.00006).

### 2.2. EGFR or EGFRvIII Overexpression Induces AR Overexpression and Nuclear Translocation, While EGFR and AKT Inhibitor Treatment Abrogates the AR Nuclear Translocation

**Figure 2:**
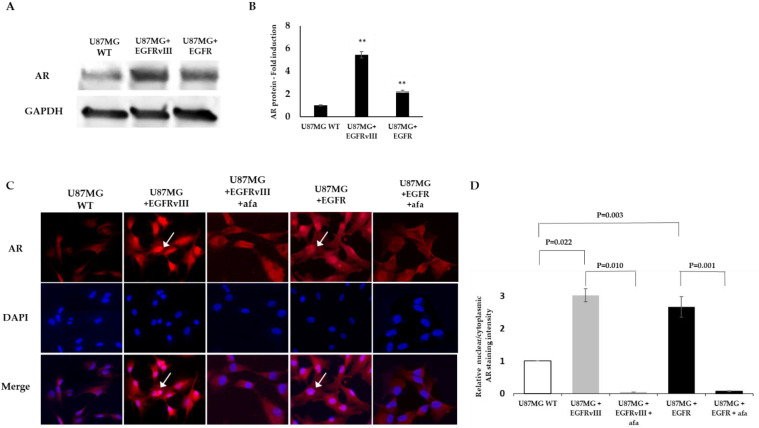
EGFR promotes AR overexpression in the U87MG glioblastoma cell line model. (**A**) Western blot analysis using anti-AR (D6F11) in U87MG WT, U87MG EGFR, and U87MG EGFRvIII cells (upper gel). The anti-GAPDH housekeeping protein was used as a loading control (lower gel). (**B**) Androgen receptor protein level in U87MG WT, U87MG EGFR, and U87MG EGFRvIII cells (*x*-axis) was analyzed by PathScan ^®^ Total Androgen Receptor Sandwich ELISA Kit. AR fold induction compared to U87MG WT is represented (*Y*-axis). The experiments were repeated three times. The results of the relative fold induction are presented as the mean ± SD. ** *p* < 0.01 versus the U87MG control group. (**C**) Immunofluorescence staining for anti-AR (D6F11) (red) in vehicle and afatinib-treated U87MG WT, EGFR, or EGFRvIII cells. Cell nuclei were stained with DAPI (blue). White arrows indicate AR nuclear staining. (**D**) Relative AR nuclear localization was determined by quantification of the relative fluorescent intensity ratio between nuclear and cytosolic compartments using ImageJ software. The results are expressed as mean ± SD following normalization to U87MG WT; *p*-values between the groups are indicated.

We tested AR protein expression in U87MG EGFR or EGFRvIII cells compared to the U87MG WT cells using an anti-AR antibody by Western blot analysis and enzyme-linked immunosorbent assay (ELISA). Densitometry of the Western blot bands revealed that the U87MG EGFR or EGFRvIII subclones had 2.8- and 5.2-fold increased AR protein levels, respectively, compared to the WT U87MG (Figure 2A). A similar result was obtained using ELISA methodology (2.2- and 5.5-fold increased for EGFR or EGFRvIII subclones, respectively) (Figure 2B).

Immunofluorescence staining using the same AR antibody that was used for the Western blot confirmed this result. U87MG EGFRvIII and U87MG EGFR cells had an intense AR staining. Both subclones had a nuclear translocation of 3.02 ± 0.2 and 2.66 ± 0.32-fold increase, respectively, compared to the wt cells. The control cells had only faint staining and a basal nuclear translocation (Figure 2C). After 6 h treatment with 5 µM afatinib, an irreversible EGFR kinase inhibitor, the AR nuclear translocation, was abrogated in U87MG EGFR by 95% and in EGFRvIII cells by 93% (Figure 2C).

### 2.3. Phospho-Kinase Array Evaluation of EGFR Downstream Effectors

The above results demonstrate that an EGFR inhibitor abrogated EGFR- or EGFRvIII-induced AR nuclear translocation, implying that EGFR can stimulate AR signaling in glioblastoma cells. To explore the potential mediators downstream of EGFR that might be involved in AR regulation, we studied the phosphorylation of 43 human kinases (Proteome Profiler Human Phospho-Kinase Array) in the U87MG WT cells and the EGFR U87MG cells before and after afatinib treatment.

**Figure 3:**
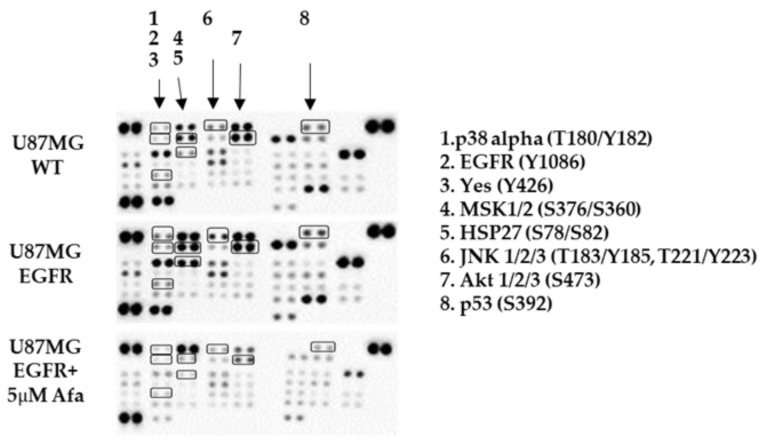
Phospho-kinase array evaluation of EGFR downstream effectors. A human phospho-kinase blot array of U87MG control (WT) cells, U87MG EGFR cells, and U87MG EGFR cells with afatinib (afa) treatment is shown. The kinases circled and numbered in the blots and either on the right of the image represent major signaling protein targets of EGFR activation.

The array revealed that following EGFR overexpression, several kinases had increased phosphorylation levels (>1.5-fold). The phosphorylation levels of eight of those kinases were decreased following afatinib treatment (>1.5-fold). [Mitogen-activated protein kinase 14 (p38 alpha) (T180/Y182); Heat shock protein 27 (Hsp27) (S78/S82); Yamaguchi Sarcoma Oncogene Homolog (Yes) (Y426); c-Jun N-terminal kinase(JNK1/2/3) (T183/Y185, T221/Y223); mitogen- and stress-activated kinases 1 and 2 (MSK1/2)(S376/S360); Tumor protein P53 (p53) (S392); AKT1/2/3 (S473);EGFR (Y1086)] (Figure 3; Table 1).

### 2.4. MK2206 Modulates EGFR-Induced AR Expression and Nuclear Translocation

**Figure 4:**
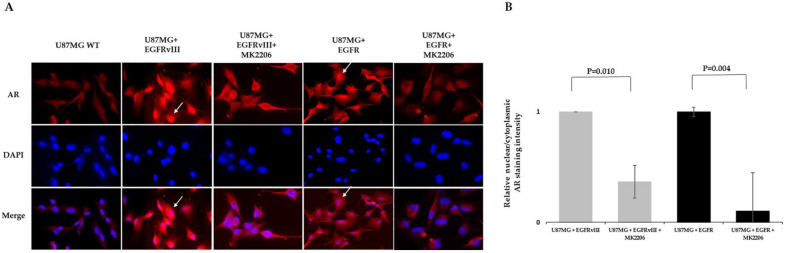
AKT inhibitor modulates EGFR-induced AR expression and nuclear translocation in the U87MG glioblastoma cell line model. (**A**) Immunofluorescence staining of AR in U87MG subclones following treatment with AKT kinase inhibitor. Immunofluorescence staining of anti-AR (D6F11) (red) in U87MG WT EGFR or EGFRvIII following 6 h treatment with vehicle or 5 µm MK2206. DAPI staining indicates the location of the nucleus (blue). Cell nuclei were stained with DAPI (blue). White arrows indicate AR nuclear staining. (**B**) Relative AR nuclear localization was determined by quantification of the relative fluorescent intensity ratio between nuclear and cytosolic compartments using ImageJ software. The results are expressed as mean ± SD following normalization to the vehicle-treated subclones, *p*-values between the groups are indicated.

pAKT^S473^ was one of the seven kinases downstream of EGFR over-phosphorylated by EGFR overexpression in an afatinib-sensitive manner. Since AKT is a known secondary messenger that plays a role downstream of EGFR signaling in glioblastoma, we explored whether AKT is involved in EGFR-induced AR regulation. For that purpose, we treated U87MG subclones with the AKT kinase allosteric inhibitor MK2206 for 6 h. This AKT inhibitor was chosen because it directly inhibits AKT1/2/3 phosphorylation at S473 and showed efficacy in glioblastoma [23]. MK2206 reduced AR nuclear translocation in U87MG EGFR by 89% and in EGFRvIII cells by 63% (Figure 4).

### 2.5. EGFR Probably Induces AR Phosphorylation on S210/213 through AKT S473

**Figure 5:**
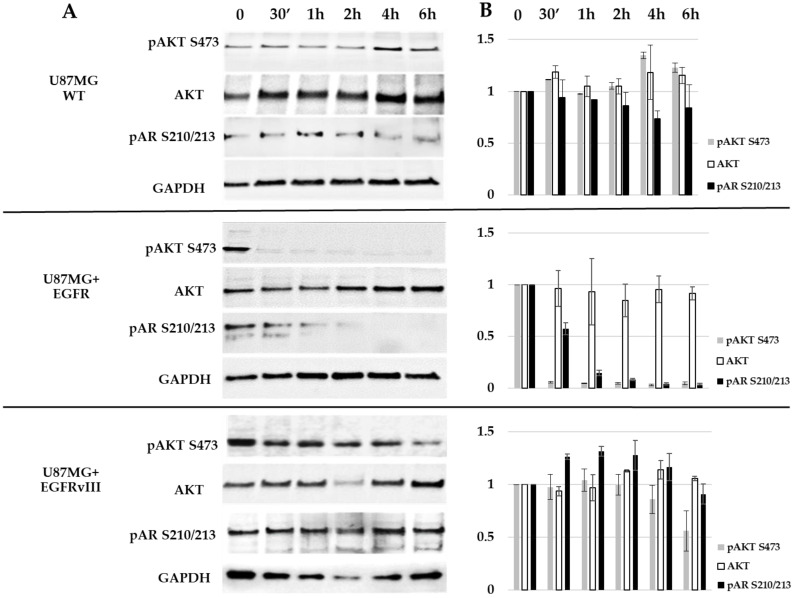
Western blot analysis of AR S210/213 and AKT S473 in U87MG subclones. (**A**) Cells were treated for 30 min (30′) to 6 h with 5 μM afatinib and analyzed with Western blotting using antibodies against AKT, pAKT (S473), and pAR (S210/213). The anti-GAPDH housekeeping protein was used as a loading control. (**B**) Protein fold change (Y-axis) of each sample compared to treatment at time zero was calculated according to band densitometry analysis with ImageJ software, following normalization to GAPDH.

Several pieces of evidence from breast and prostate cancers show that AKT S473 can phosphorylate AR at S210/213 [24,25,26,27,28,29,30,31]. Based on the earlier results, we explored the effect of afatinib on AKT S473 and AR S210/213. To that end, U87MG WT and EGFR or EGFRvIII cells were treated for 30 min to 6 h with 5 µM afatinib; their cell lysates were analyzed with Western blotting (Figure 5). AR phosphorylation at S210/213 was detected at time 0 in all U87MG subclones, although it was detected at lesser levels in the U87MG WT cells. Similar to the array results, afatinib treatment abolished AKT phosphorylation (S473) in U87MG EGFR cells 30 min after treatment (reduction of 95%), while AR phosphorylation was gradually abolished between 30 min and 2 h of treatment (reduction of 42–91% respectively) and was completely abolished 4 h following treatment (Figure 5). However, the U87MG EGFRvIII cells had no reduction in AR phosphorylation and 46% reduction in AKT phosphorylation at 6 h (Figure 5). Afatinib affected neither AKT phosphorylation nor AR phosphorylation in the U87MG WT cells (Figure 5).

**Figure 6:**
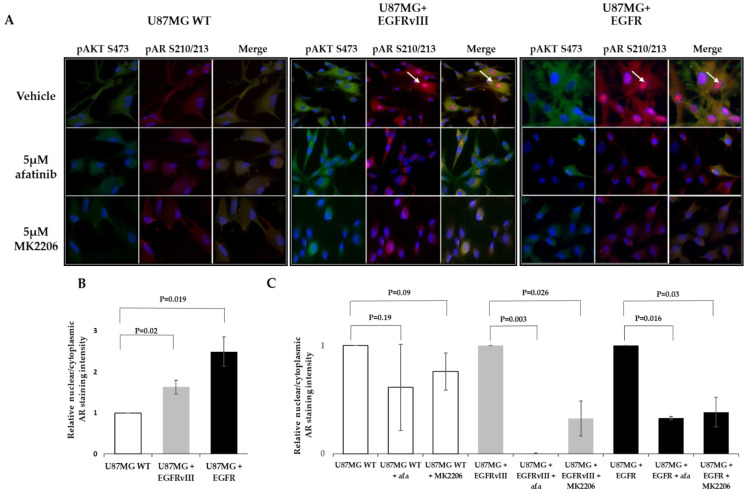
Immunostaining of AR S210/213 and AKT S473 in U87MG subclones. (**A**) U87MG WT, U87MG EGFR, and U87MG EGFRvIII cells were treated with vehicle (1% DMSO), 5 μM afatinib, or 5 μM MK2206 for 6 h and analyzed by immunofluorescence with antibodies against pAR (S210/213) (red) and pAKT (S473) (green). (**B**,**C**) AR nuclear localization was determined by quantifying the relative fluorescent intensity ratio between nuclear and cytosolic compartments using ImageJ software. The results are expressed as mean ± SD following normalization to U87MG WT (**B**) or the vehicle-treated subclones (**C**); *p*-values between the groups are indicated.

To confirm the Western blotting results and explore either pAR^S210/213^ nuclear translocation and/or physical interaction between pAKT^S473^ and pAR^S210/213^, the three U87MG subclones were analyzed with immunofluorescence. The cells were treated for 6 h with 5 µM afatinib, 5 µM MK2206, or vehicle (1% dimethyl sulfoxide (DMSO)) and then stained with anti-pAR^S210/213^ and -pAKT^S473^ antibodies. As the Western blot analysis demonstrated, pAR^S210/213^ was highly visualized in the vehicle-treated EGFR and EGFRvIII cells compared to the WT cells, in which very low levels were observed. It should be mentioned that in the EGFRvIII subclone, pAR^S210/213^ was visualized only in about 20% of the cells. Afatinib and MK2206 had almost no effect on the pAR^S210/213^ phosphorylation levels in the WT cells. However, it reduced the pAR^S210/213^ staining in U87MG EGFR and EGFRvIII clones (Figure 6). EGFR and EGFRvIII induced pAR^S210/213^ nuclear translocation (2.5 and 1.6-fold vs. WT cells, respectively) (Figure 6A,B). Nuclear translocation was decreased in EGFR and EGFRvIII following afatinib by 67% and 99%, respectively, and it decreased by 61% and 67% after MK2206 treatment. Cytoplasmic pAKT^S473^ staining was highly visualized in the vehicle-treated EGFR and EGFRvIII cells. Low levels of pAKT^S473^ were detected in the WT cells and afatinib-treated EGFR cells. Afatinib had almost no effect on pAKT^S473^ levels in the WT cells (Figure 6) and had a moderate effect on pAKT^S473^ levels in the EGFRvIII cells (Figure 6). In all the subclones, a significant pAKT^S473^ and pAR^S210/213^ colocalization was detected (Figure 6, Table 2).

### 2.6. Afatinib and Enzalutamide Combination Therapy Yields Improved Efficacy in Glioblastoma Cell Lines

**Figure 7:**
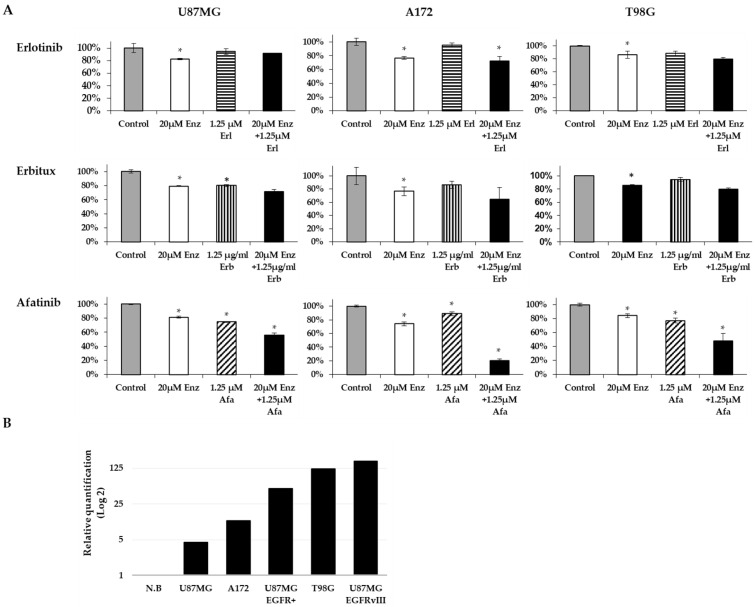
The combination effect of an AR antagonist and EGFR inhibitors on glioblastoma cell lines. (**A**) U87MG, A172, and T98G glioblastoma cells were treated for 72 h with EGFR inhibitors (striped columns): erlotinib (Erl), erbitux (Erb), afatinib (Afa), or the AR antagonist, enzalutamide (Enz; white columns), alone or in combination (black columns). Cell density is expressed as the percentage of cells treated with vehicle (gray columns). All experiments were repeated at least three times. The results are the mean ± SD. * *p* < 0.05 indicates significant results of each treatment compared to vehicle-treated cells (Student’s two-tailed *t*-test). (**B**) qPCR analysis results of the HPRT-normalized relative quantification of EGFR mRNA expression in the glioblastoma cells versus expression in a commercial RNA mixture of 23 normal brains (N.B).

As mentioned above, we have previously shown [13] that inhibiting AR signaling led to glioblastoma cell death, suggesting that AR is essential for the cellular survival of glioblastoma. Accordingly, we explored whether inhibiting EGFR signaling would stimulate AR antagonist-mediated cell death. We studied whether combining EGFR kinase inhibitors with AR antagonists would induce synergistic efficacy in glioblastoma. The cell viability of three glioblastoma cell lines (U87MG, A172, T98G) was studied at 72 h following treatment with EGFR kinase inhibitors combined with enzalutamide (AR antagonist) or as monotherapy. Erlotinib and erbitux had very low efficacy in all cell lines as monotherapy or combined with enzalutamide. In contrast, afatinib had a superior effect as combination therapy in all cell lines (the combination effect was analyzed with the coefficient of drug interaction (CDI)) (Figure 7A). Afatinib (1.25 µM) plus 20 µM enzalutamide had an additive effect on U87MG cell viability (CDI = 0.95), while it had a synergistic effect in the A172 and T98G cells (CDI = 0.31 and 0.74, respectively).

It should be noted that A172 and T98G had EGFR overexpression, while U87MG cells had lower EGFR levels (Figure 7B).

**Figure 8:**
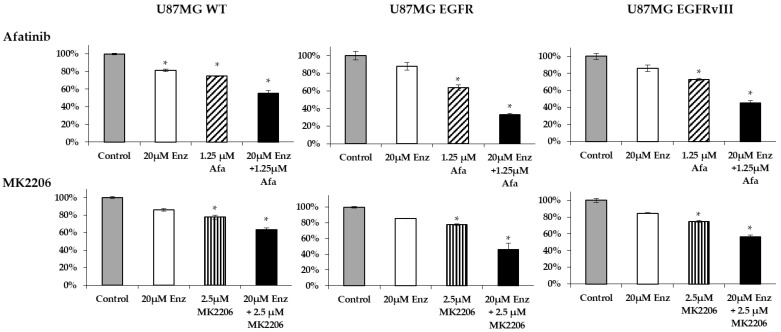
The combination effect of an AR antagonist with EGFR/AKT inhibitor in U87MG subclones. U87MG WT, U87MG EGFR, and U87MG EGFRvIII cells were treated for 72 h with 1.25 μM afatinib (Afa) (striped columns) or 20 μM enzalutamide (Enz; white columns) alone or in combination (black columns); 2.5 μM MK2206 (striped columns) or 20 μM enzalutamide (Enz; white columns) alone or in combination (black columns). Cell density is expressed as the percentage of cells treated with vehicle (gray columns). The results are the mean ± SD. * *p* < 0.05 indicates significant results of each treatment compared to vehicle-treated cells (Student’s two-tailed *t*-test).

Guided by the above results, we studied whether the synergistic effect of the combination therapy was attributed to EGFR overexpression/activation in AR-induced cell survival. For that purpose, we tested the effectiveness of afatinib or MK2206 plus enzalutamide on U87MG EGFR or EGFRvIII cells and compared it to the results obtained for the U87MG WT cells. Unlike the additive effect of afatinib or MK2206 plus enzalutamide in the U87MG WT (CDI = 0.95 and 0.95, respectively), the combination therapy induced synergistic efficacy in the EGFR and EGFRvIII cells. In the EGFR and EGFRvIII cells, afatinib plus enzalutamide had a CDI of 0.22 and 0.28, respectively, while the CDI for MK2206 plus enzalutamide was 0.7 and 0.88, respectively (Figure 8).

## 3. Discussion

Our results demonstrate that EGFR (HER1) signaling affects AR activation in glioblastoma. EGFR or EGFRvIII overexpression induced AR overexpression and nuclear translocation. Moreover, we demonstrated that EGFR kinase inhibitor treatment abrogates AR nuclear translocation in both U87MG EGFR and EGFRvIII cells. These results are similar to those in hormone-refractory prostate cancer cells, where the forced overexpression of HER2 kinase increased AR expression through AR signaling [32,33]. The fact that the forced overexpression of EGFR family members can induce AR expression might be one reason for the correlation between AR and EGFR mRNA expression seen in our study. Yet, studies of other types of cancer have yielded conflicting results regarding the correlation between EGFR and AR expression. In gastric cancer, EGFR overexpression correlated significantly with AR overexpression [34], and a negative correlation was observed in breast cancer [35]. In prostate cancer, some have shown that AR and EGFR proteins are inversely correlated, while others have shown a positive correlation [36,37]. However, there are no published studies regarding the interplay between EGFR and AR in gliomas.

While studying the potential secondary messengers involved in the EGFR-induced AR signaling pathway, we found that EGFR overexpression stimulated a 14.5-fold induction in EGFR phosphorylation at Y1068. pEGFR^Y1068^ induction has been documented before in both U87MG EGFR and EGFRvIII subclones [38,39].

pAKT^S473^ was one of the seven kinases downstream of EGFR over-phosphorylated by EGFR overexpression-induced activation and reduced by afatinib. As breast and prostate cancers have yielded much evidence that pAKT^S473^ can phosphorylate AR at S210/213 [24,25,26,27,28,29,30,31], we examined whether this was also the case in gliomas. pAKT^S473^ and pAR^S210/213^ immunostaining revealed that pAKT^S473^ colocalized with pAR^S210/213^ in all U87MG subclones. When MK2206 inhibited AKT kinase activity, pAR^S210/213^ levels were reduced, and its nuclear translocation was abrogated. That implies that pAKT^S473^ directly phosphorylates AR at S210/213, as described in prostate cancer [26,40]. In prostate cancer, pAR^S210/213^ is responsible for AR protein stability and nuclear translocation [41].

Western blotting demonstrated that in the u87MG EGFR, AKT S473 phosphorylation was abolished at 30 min following afatinib treatment, while AR phosphorylation was gradually decreased between 30 min and 2 h of treatment and was abolished at 4 h following treatment. These results strengthen the hypothesis that AKT kinase acts in AR phosphorylation at S210/213 in gliomas and implies that the decreased pAR^S210/213^ is attributed to the inhibitory effect of afatinib on pAKT^S473^. This result agrees with the results of others, where PI3K–AKT signaling activation augmented S210/213 phosphorylation of AR in different cell lines [25,28]. Furthermore, AKT activation by PI3K increases pARS210/213 phosphorylation in prostate cancer, and the PI3K inhibitor LY294002 suppressed it [26]. These findings are relevant to glioblastoma not only because most glioblastomas have *EGFR* alteration but also because at least 60% of the glioblastomas have PI3K signaling pathway deregulation resulting from genetic alterations in the *PTEN* tumor suppressor gene on 10q23 at the level of loss of heterozygosity, mutation, and methylation [42].

In the U87MG EGFRvIII clone, pAR^S210/213^ was visualized only in about 20% of cells; either afatinib or MK2206 decreased this staining. Furthermore, although pARS210/213 nuclear staining abrogated after afatinib treatment, a cytoplasmic pAKT^S473^ and pAR^S210/213^ staining were still evident. This result is similar to another study that showed only a minor reduction in pAKT^S473^ in U87MG EGFRvIII cells after afatinib treatment [26]. The above results might explain the results of the Western blot analysis, which demonstrated only a minor inhibitory effect on pAKT^S473^ and pAR^S210/213^ at 6 h following afatinib treatment. There is a discrepancy between the results gained with the full-length AR antibody immunostaining and the pAR^S210/213^ in U87MG EGFRvIII. While a robust AR nuclear staining was demonstrated with the anti-AR monoclonal antibody (D6F11), only 20% nuclear staining was observed with the phosphospecific anti- pAR^S210/213^. One hypothesis for those conflicting results is that AR activation in EGFRvIII-expressing cells is partly achieved by AR phosphorylation at additional residues, by alternative signaling pathways, as known from prostate cancer [43]. This hypothesis should be further studied in future research.

Based on our results for the interplay between EGFR and AR signaling, we assumed that inhibiting EGFR-induced AR signaling upstream and downstream of AR would be beneficial. This assumption was proven by the synergistic effect results obtained by combining an EGFR/AKT kinase inhibitor with an AR antagonist on U87MG EGFR and U87MG EGFRvIII cells. A similar result was seen in triple-negative breast cancer, where concomitant administration of the anti-androgen bicalutamide with an EGFR inhibitor had an anti-proliferative effect [44].

## 4. Materials and Methods

### 4.1. Patients and Tumors

RT quantitative real-time PCR mRNA study was performed on 28/32 cDNAs synthesized previously [13] from newly diagnosed primary glioblastoma samples from adults of both sexes. The molecular parameters of these patients are listed in [13]. The cDNA of patients’ numbers 20, 28, 31, and 32 used in our previous study were not available.

The primers used for studying the expression of the specified genes are listed (Table 3).

### 4.2. RNA Extraction, cDNA Preparation, and qPCR

RNA extraction, cDNA preparation, and qPCR were performed as previously described [13].

### 4.3. Cell Culture and Treatments

Human A172, U87MG, and T98G glioblastoma cell lines were purchased from ATCC [13]. U87MG WT, EGFR-overexpressing (U87MG EGFR), and EGFRvIII-overexpressing cells (U87MG EGFRvIII) were generated as described previously [45]. The cells were maintained in DMEM with 10% FBS and 1× pen/strep/glutamine supplemented with various selected antibiotics. G418 (500 μg/mL, Invivogen, Toulouse, France) was included for the U87MG EGFRvIII cells; 60 µg/mL hygromycin (Gold Biotechnology, Inc., 1328 Ashby Rd, Olivette, MO 63132, United States) was added for the U87MG EGFR cells.

Cells (1 × 10^4^) were plated in triplicate in 24-well plates (Nunc ™, Thermo Fisher Scientific, Rochester, NY, USA ) or 8-cell chamber glass slides (Ibidi, Martinsried, Lochhamer Schlag 11, 82166 Gräfelfing, Germany) and allowed to attach overnight. The next day, the cells were treated with the following: AR inhibitor, enzalutamide (A2S Technologies, Gan Rave, Israel); EGFR inhibitors: afatinib (A2S Technologies, Gan Rave, Israel), erlotinib (Roche Pharmaceuticals Ltd., Hod Hasharon, Israel), or erbitux (Merck, Darmstadt, Germany); or AKT inhibitor: MK2206 (MedChemExpress,1 Deerpark Dr # Q, Monmouth Junction, NJ 08852, United States).

### 4.4. Cell Survival and Drug Interaction Analysis

Cell density was measured with the crystal violet dye binding assay as described previously [13]. Briefly, 0.5% crystal violet (Sigma-Aldrich, 3300 S 2nd St #3306, St. Louis, MO 63118, United States) was added to each well following fixation with 4% paraformaldehyde (Gadot, Netanya, Israel); the dye was solubilized with 10% acetic acid (Gadot) and read at 590 nm in a DTX 880 Multimode detector microplate reader (Beckman Coulter, Nyon, Switzerland). The percentage of cell density was calculated based on the average absorbance of the treated samples compared to that of the control. The combination effect was analyzed with the coefficient of drug interaction (CDI). The CDI value was determined as follows: CDI = EAB/(EA × EB), where EAB is the OD ratio of the combination group and control group, EA is the OD ratio of drug A and the control group, and EB is the OD ratio of drug B and the control group. If CDI < 1, the two drugs were synergistic; if CDI = 1, the two drugs were additive; if CDI > 1, the two drugs were antagonistic.

### 4.5. Immunofluorescence Staining, Western Blotting, and ELISA

For immunofluorescence, cells were fixed in 4% paraformaldehyde for 10 min at room temperature and permeabilized with cold methanol for 20 min. Then, cells were washed twice with PBS-T and incubated in blocking solution (3% BSA, 0.1% Tween in PBS) for 30 min, which was followed by a 60 min incubation at 37 °C with one of the following primary antibodies: anti-AR (D6F11; 1:100, Rabbit mAb, Cell Signaling Technology, Danvers, Massachusetts, United States), anti-pAKT473 (ab81283; 1:100; Abcam, Cambridge, United Kingdom), and anti-pAR S210/213 (1:100; cat. no. ab45089; Abcam). Then, the cells were incubated for 60 min at room temperature with the relevant secondary antibody: Alexa Fluor 488-conjugated anti-rabbit IgG (A21206; 1:200; Invitrogen, Waltham, MA, USA) or Alexa Fluor 555-conjugated anti-mouse IgG (ab150118; 1:200; Abcam). The nuclei were stained with DAPI (D9542, Sigma-Aldrich); the cells were visualized and captured with an Olympus BX3 Upright Microscope using cellSens Dimension imaging software. An average of 5 fields with approximately 10 cells per field from three independent experiments was captured for each group, and image analysis was carried out using the NIH Fiji/ImageJ. The integrated density (IntDen) measurements of the color threshold tool (image > adjust > color threshold) were used to calculate nuclear localization. Analysis of nuclear localization of AR was done by calculating the relative ratios of the nucleus to cytosol intensity. Colocalization and subsequent Pearson’s coefficient calculation between AR S210/213 and AKT s473 were performed using the Coloc2 plugin in Fiji [46]. For Western blotting and Elisa assay, the cells were lysed with Buffer 6 (cat. no. 895561, R&D Systems, Minneapolis, MN, USA,) supplemented with protease and phosphatase inhibitor (ab201120; Abcam). Total protein concentration was determined using a BCA quantification assay kit (Cell Signaling Technology). Western blotting was performed as previously described [13], using the antibodies used for immunofluorescence and GAPDH (0411, 1:1000: Santa Cruz Biotechnologies, 2145 Delaware Ave, Santa Cruz, CA 95060, United States), as indicated. Protein fold change of each sample compared to treatment at time zero was calculated according to band densitometry analysis with ImageJ software, following normalization to GAPDH.

For AR ELISA assay, 100 μg of total protein for each cell lysate (same as above) were incubated in a 96-well Androgen Receptor ELISA plate (PathScan Total Androgen Receptor Sandwich ELISA Kit 12850, Cell Signaling Technology) to determine the AR relative levels. The assay was performed following the manufacturer’s instructions. The spectrophotometric determination was done by reading the absorbance at 450 nm in a DTX 880 Multimode detector microplate reader. Data were analyzed by using Multimode Analysis Software.

### 4.6. Human Phospho-Kinase Antibody Array

The protein lysates from 5,000,000 U87MG WT, U87MG EGFR, or U87MG EGFR have been treated with 5 μM afatinib for 6 h. The relative phosphorylation levels of 43 kinase phosphorylation sites were determined using the human phospho-kinase array kit (ARY003B, R&D Systems, Minneapolis, MN, USA) following the manufacturer’s instructions. Briefly, the Proteome Profiler Human Phospho-Kinase Array Kit is a membrane-based sandwich immunoassay. Cell lysates were incubated overnight with the Human Phospho-Kinase Array. Capture antibodies spotted in duplicate on nitrocellulose membranes bind to specific target proteins present in the sample. Then, the membrane was washed to remove unbound proteins, and the phosphorylation of captured proteins was detected with biotinylated phospho-specific detection antibodies. Streptavidin–HRP and chemiluminescent detection reagents were applied to produce a signal at each capture spot corresponding to the amount of phosphorylated protein bound.

The chemiluminescent signal was acquired using the blot documentation system Fusion Solo (Vilber, Collégien, France) and was analyzed using the ImageJ software (National Institutes of Health, Bethesda, MD, USA) [47]. The pixel density of each spot was calculated following subtraction of the background (negative control) and normalization to the positive control spots.

### 4.7. Statistical Analysis

Student’s two-tailed *t*-test was used to determine the mean differences between the two groups. *p* < 0.05 is considered significant. Data are presented as mean ± SD. The correlation was calculated in accordance with Spearman’s rank correlation coefficient.

## 5. Conclusions

EGFR signaling is involved in AR activation in glioblastoma. Our results support combination therapy involving an EGFR inhibitor with an AR antagonist, warranting further investigation.

## Figures and Tables

**Table 1:** The kinases with high fold phosphorylation levels in response to overexpression of EGFR that were decreased following afatinib treatment.

Kinase	U87MG Control Pixels Densitometry	U87MG EGFR Pixels Densitometry	EGFR-Derived Fold Induction	U87MG EGFR + Afatinib Pixels Densitometry	Afatinib-Derived Fold Reduction
**EGFR (Y1086)**	14	203	**14.5**	1	**203**
**Yes (Y426)**	117	397	**3.4**	1	**397**
**p53 (S392)**	1406	2412	**1.7**	682	**3.5**
**p38 alpha (T180/Y182)**	7	904	**129**	174	**5.2**
**HSP27 (S78/S82)**	381	6336	**17**	1	**6336**
**MSK1/2 (S376/S360)**	4191	8184	**1.95**	525	**15.6**
**AKT 1/2/3 (S473)**	7060	10,753	**1.5**	1015	**10.6**
**JNK 1/2/3 (T183/Y185, T221/Y223)**	774	2305	**3**	341	**6.8**

**Table 2:** Correlation analysis of the colocalization of AR S210/213 with AKT S473 in the U87MG subclones.

Treatment		U87MG WT	U87MG EGFRvIII	U87MG EGFR
**Vehicle**	Pearson correlation R values	0.98	0.94	0.87
	Pearson Correlation *p*-value	<0.00001	<0.00001	0.010899
**5 μM Afatinib**	Pearson correlation R values	0.99	0.464	0.86
	Pearson Correlation *p*-value	0.00015	0.000037	0.017
**5 μm MK2206**	Pearson correlation R value	0.834	0.62	0.836
	Pearson Correlation *p*-value	0.009546	<0.00001	0.000026

**Table 3:** The primers that were used to study the expression of the specified genes.

Gene	Primer Sequence
***AR***	F: ACCGAGGAGCTTTCCAGAATC
	R: AGGCTCTGGGACGCAACCT
***EGFR***	F: GCGTCTCTTGCCGGAATGT
	R: CTTGGCTCACCCTCCAGAAG
**TATA-binding protein (*TBP*)**	F: CCACTCACAGACTCTCACAAC
	R: CTGCGGTACAATCCCAGAACT
**Hypoxanthine phosphoribosyltransferase 1 (*HPRT*)**	F: GATGGTCAAGGTCGCAAGC
	R: ATATCCTACAACAAACTTGTCTGGAA
